# ErbB2_pY_ _‐1248_ as a predictive biomarker for Parkinson's disease based on research with RPPA technology and in vivo verification

**DOI:** 10.1111/cns.14407

**Published:** 2023-08-11

**Authors:** Meng Jin, Ruidie Shi, Daili Gao, Baokun Wang, Ning Li, Xia Li, Attila Sik, Kechun Liu, Xiujun Zhang

**Affiliations:** ^1^ Biology Institute, Qilu University of Technology (Shandong Academy of Sciences) Ji'nan China; ^2^ Engineering Research Center of Zebrafish Models for Human Diseases and Drug Screening of Shandong Province Ji'nan China; ^3^ School of Psychology North China University of Science and Technology Tang'shan China; ^4^ Mills Institute for Personalized Cancer Care, Fynn Biotechnologies Ltd. Ji'nan China; ^5^ Institute of Transdisciplinary Discoveries, Medical School University of Pecs Pécs Hungary; ^6^ Institute of Clinical Sciences, Medical School University of Birmingham Birmingham UK; ^7^ Institute of Physiology, Medical School University of Pecs Pécs Hungary

**Keywords:** ErbB2, mouse, PD, reverse phase protein array, SH‐SY5Y, zebrafish

## Abstract

**Aims:**

This study aims to reveal a promising biomarker for Parkinson's disease (PD) based on research with reverse phase protein array (RPPA) technology for the first time and in vivo verification, which gains time for early intervention in PD, thus increasing the effectiveness of treatment and reducing disease morbidity.

**Methods and Results:**

We employed RPPA technology which can assess both total and post‐translationally modified proteins to identify biomarker candidates of PD in a cellular PD model. As a result, the phosphorylation (pY‐1248) of the epidermal growth factor receptor (EGFR) ErbB2 is a promising biomarker candidate for PD. In addition, lapatinib, an ErbB2 tyrosine kinase inhibitor, was used to verify this PD biomarker candidate in vivo. We found that lapatinib‐attenuated dopaminergic neuron loss and PD‐like behavior in the zebrafish PD model. Accordingly, the expression of ErbB2_pY‐1248_ significantly increased in the MPTP‐induced mouse PD model. Our results suggest that ErbB2_pY‐1248_ is a predictive biomarker for PD.

**Conclusions:**

In this study, we found that ErbB2_pY‐1248_ is a predictive biomarker of PD by using RPPA technology and in vivo verification. It offers a new perspective on PD diagnosing and treatment, which will be essential in identifying individuals at risk of PD. In addition, this study provides new ideas for digging into biomarkers of other neurodegenerative diseases.

## Graphical abstract

We employed reverse phase protein array technology which can assess both total and post‐translationally modified proteins to identify biomarker candidates of Parkinson's disease (PD) in a cellular PD model. As a result, the phosphorylation (pY‐1248) of the epidermal growth factor receptor (EGFR) ErbB2 may be a promising biomarker for PD. In addition, lapatinib, an ErbB2 tyrosine kinase inhibitor, was used to verify this PD biomarker candidate in vivo. We found that lapatinib attenuated dopaminergic neuron loss and PD‐like behavior in the zebrafish PD model. Our results suggest that ErbB2_pY‐1248_ is a predictive biomarker for PD, which gains time for early intervention in PD, thus increasing the effectiveness of treatment and reducing disease morbidity.

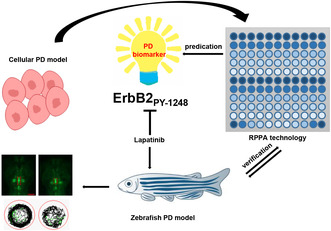

## 1 INTRODUCTION

Parkinson's disease (PD) is the second most common neurodegenerative disorder, which prevalence increases steadily with age.^1^ Its main clinical manifestations are resting tremor, myotonia, postural dysregulation, and bradykinesia.^2^ In severe cases, PD is accompanied by memory impairments and other dementing symptoms.^3^ The neuropathological hallmark of PD is the loss of dopaminergic neurons in the substantia nigra and the formation of intraneuronal proteinaceous inclusions, called Lewy bodies, which are mainly composed of α‐synuclein.^4^, ^5^ An important limitation of PD therapy is the delayed diagnosis and therapy. Biomarkers of PD with high sensitivity and specificity are urgently needed to facilitate early diagnosis of PD, detect disease progression, and assess response to existing and future treatments.

The gold standard for diagnosing PD is autopsy neuropathology. Due to the limitations of studies using the human brain, researchers have developed various methods that use both in vivo and in vitro experiments to model various aspects of PD. Current experimental models of PD are divided into two categories: neurotoxic and genetic.^6^ PD neurotoxic model mainly induced by MPTP or 6‐OHDA. 1‐Methyl‐4‐phenyl‐1,2,3,6‐tetrahydropyridine (MPTP) is one common neurotoxin used in generating animal PD models. MPTP enters the brain, converted to the toxic 1‐methyl‐4‐phenylpyridinium (MPP^+^).^7^ MPP^+^ reduces dopamine synthesis by inhibiting the activity of tyrosine hydroxylase (TH) in neurons.^8^ The pathology of PD is characterized by the loss of dopaminergic neurons in the substantia nigra due to oxidative stress and partially susceptible genes leading to a lower dopamine level. This lowering in dopamine level further contributes to the characteristic motor impairments. Several studies have supported the role of dopamine in PD.^9^, ^10^ 6‐hydroxydopamine (6‐OHDA) can be actively taken into the cell by membrane transporters on dopamine neuronal terminals or soma, which selectively act on dopaminergic neurons, leading to neuronal degeneration and death, causing PD symptoms. 6‐OHDA is usually used in a cellular model for PD research and is widely used in anti‐PD drug screening studies. It has been reported that PD‐related genes, including *SNCA*, *LRRK2*, *PINK1*, *PARKIN*, and *DJ‐1* are used in the generation of transgenic animal PD models.^11^, ^12^ Studies have shown that *Pink1* knockout mice induce symptoms, which is similar to PD penitents, including dopamine reduction and decreased locomotor activity.^13^ In addition, induced pluripotent stem cells (iPSCs) from marmosets with the G2019S mutation in the *LRRK2* gene show PD‐like symptoms.^14^

Given the structural similarities between the zebrafish dopamine neuron system and the human striatum, zebrafish has become an excellent model for neurodegenerative and neuropharmacological studies.^15^, ^16^ Some studies applied the MPTP‐induced zebrafish PD model to characterize PD‐like cognitive deficits by assessing spatial working memory and spontaneous alternation behavior.^17^, ^18^ MPTP‐induced zebrafish PD model is used to study the mitochondria function in the berberine's neuroprotective activity.^19^ It has been reported that *Ganoderma lucidum* extracts and curcumin could improve motor impairments and reduce dopaminergic neuron loss by using the mouse PD model.^20^, ^21^ SH‐SY5Y cells are human neuroblastoma cells with many features of neurons, which have been widely used as dopaminergic neuronal model cells for PD research.^22^, ^23^, ^24^, ^25^ 6‐OHDA‐induced neurotoxicity on SH‐SY5Y cells is considered the most widely used in vitro model that causes an accumulation of reactive oxygen species near the cells themselves that mimics dopaminergic striatal neurodegeneration in PD.^26^, ^27^, ^28^, ^29^

ErbB proteins belong to subclass I of the receptor tyrosine kinase superfamily. There are four members of the ErbB family: ErbB1/HER1, ErbB2/Neu/HER2, ErbB3/HER3, and ErbB4/HER4. Ligand binding to ErbB receptors induces the formation of receptor homo‐and heterodimers and the activation of intrinsic kinase domains, leading to the phosphorylation of specific tyrosine residues within the cytoplasmic tail. These phosphorylated residues serve as docking sites for a range of proteins whose recruitment activates intracellular signaling pathways.^30^, ^31^, ^32^ ErbB2 has several autophosphorylation sites, including tyrosines1248, 1221, 1222, 1139, 1196, and 1112. Phosphorylation at these sites may reflect ErbB2 activity.^33^ One of the most widely studied phosphorylation sites is tyrosine1248, the only site for which an antibody is established to be stable in human tissues.^34^, ^35^, ^36^, ^37^, ^38^ Lapatinib is a small‐molecule tyrosine kinase inhibitor of ErbB1 and ErbB2, which can reversibly inhibit ErbBs, blocking their phosphorylation.^39^, ^40^ It has been reported that dysregulation of the ErbB system may contribute to the pathogenesis of many brain disorders, including Alzheimer's disease, epilepsy, and PD.^41^, ^42^ Studies also have shown the association between variants of the ErbB2 gene with PD.^43^

Reverse phase protein array (RPPA) has emerged as an effective high‐throughput method for targeted proteomics, allowing quantification of protein expression profiles in large sample sets while requiring shallow biological sample volumes.^44^ This technique can be applied to many fields, including quantitative protein profiling of cells or tissues, drug screening, and target studies, pharmacodynamic analysis, and personalized therapy. Notably, RPPA is particularly useful for identifying biomarkers of diagnostic, prognostic, and therapeutic responses.^45^, ^46^, ^47^, ^48^

In this study, we employed RPPA to predict the potential biomarkers of PD, followed by in vivo validation. Our study revealed a promising biomarker for PD, which provides a new way to detect PD at an early stage as well as is essential for improving diagnosis and helping monitor disease progression.

## 2 MATERIALS AND METHODS

### 2.1 Animals and maintenance

According to standard procedures, wild‐type *AB* zebrafish and the transgenic zebrafish *vmat2: GFP* were maintained. Adult zebrafish were maintained at a constant temperature of 28.5°C on a constant photoperiod (14 h bright/10 h dark). The water was circulated continuously, feeding twice a day with commercial flake fish food supplemented with live brine shrimp. Zebrafish embryos were obtained from the natural mating of adult zebrafish and raised and maintained in an incubator at 28 ± 0.5°C.

Male C57BL/6 mice (age 8 weeks, weighing 20–30 g) were purchased from Jiangsu Huachuang Xinnuo Pharmaceutical Technology Co., Ltd and were housed in a temperature and humidity‐controlled environment with a 12/12 h light/dark cycle. After 1 week of acclimation, mice were randomized into two groups: the Ctl group and the MPTP group. Mice were intraperitoneally injected with 30 mg/kg/day MPTP (dissolved in 0.9% saline) for seven consecutive days to establish a mouse PD model.

### 2.2 Cell culture and treatment

SH‐SY5Y cells were cultured in DMEM medium supplemented with 10% fatal bovine serum (C04001500, VivaCell Bioscience) and 1% penicillin–streptomycin (C3420‐0100, VivaCell Bioscience) in an incubator containing 5% CO_2_ at 37°C. For the following experiments, cells were divided into the following groups and treated for 6 h: Control (Ctl) group (medium only), and 6‐OHDA‐treated group (1 mM).

### 2.3 RPPA analysis

Cells from different groups were collected in cold RIPA lysis buffer (P0013B, Beyotime Biotechnology) containing a 0.1% protease inhibitor cocktail (HY‐K0010‐1, MCE). After centrifuging at 11000*g* for 30 min at 4°C, supernatants were collected. Protein concentration was quantified with a pierce BCA protein assay kit (P0010S, Beyotime Biotechnology) and samples were adjusted to 1.5 μg/μL. Briefly, samples were manually diluted in five serial twofold dilutions with lysis buffer. They were then printed on nitrocellulose‐coated slides using an Aushon Biosystems 2470 arrayer. Slides were probed with validated primary antibodies, followed by incubation with each of the secondary antibodies Biotin conjugated‐Goat anti‐Rabbit IgG (E0432, Agilent), Goat anti‐Mouse IgG (E0433, Agilent). The signal was amplified using a Dako Cytomation‐catalyzed avidin‐biotin‐peroxidase system. Stained RPPA slides were scanned using TissueScope LE120 (Huron Digital), generating signal intensities. The SuperCurve software then processed the spots of all horizontal samples on the slides to evaluate the total protein amount present in the spotted samples. Finally, the median polish method is used to correct the protein measurements for loading. A quality control classifier assessed RPPA slide quality in the R package ‘SuperCurve’. Only slides with a quality score above 0.6 (range: 0–1) were retained for further analysis.

### 2.4 Western blot

The desired protein samples were from SH‐SY5Y cells and mouse brains. The protein concentration was measured by a BCA assay kit. The protein was denatured at 100°C for 5 min in the 5 × SDS–PAGE loading buffer (WB‐0091, Beijing DingGuo ChangSheng Biotech. Co. Ltd.). Then the equal amount of protein was subjected to 12% SDS–PAGE gel electrophoresis and transferred onto NC membranes. After blocking with 5% skim milk in TBST for 2 h at room temperature, membranes were incubated overnight at 4°C with primary antibodies against β actin (1:5000, A5441, Sigma), ErbB2_pY‐1248_ (1:1000, AF1768, R&D Systems), ErbB2 (1:400, sc‐377,344, Santa Cruz Biotechnology), and ErbB1 (1:400, 2232S, Cell Signaling Technology). The next day, these membranes were incubated with second antibodies, anti‐mouse (1:5000, ZB‐2305, Beijing ZhongShan JinQiao Biotechnology) and anti‐rabbit (1:5000, ZB‐5301, Beijing ZhongShan JinQiao Biotechnology) at room temperature for 2 h after being washed three times with 1 × TBST. After being washed three times with 1 × TBST for the membranes, the immunoreactivity bands were visualized using chemiluminescent HPR substrate (P90719, Millipore). The grayscale value of proteins was calculated and analyzed using Image‐J software.

### 2.5 Dopaminergic neurons length measurement

After being treated with MPTP (50 μM, HY‐15608, MCE) from 24 to 96 hours post‐fertilization (hpf) or 6‐OHDA (250 μM, HY‐B1081A, MCE) from 48 to 96 hpf, the transgenic zebrafish *vmat2: GFP* was placed under stereo fluorescence microscope (Zeiss), and the development of dopaminergic neurons was recorded. Siz zebrafish larvae were randomly selected for each group.

### 2.6 PD‐like behavior recording

After being treated with drugs for 120 h, the wild‐type *AB* zebrafish larvae were transferred to 48 well plates with 1 mL aquarium water for acclimatization for 10 min in ZebraBox. The locomotion activities of each larva from different experimental groups were recorded immediately by the Zeblab video‐tracking system (Viewpoint) for 20 min. The measurements were repeated three times, each with six animals per group, and analyzed by Zeblab software (Viewpoint).

### 2.7 Rotarod test

Mice were placed on an accelerating rotating rod with a speed increased from 1 to 50 rpm. It gradually increased during the trial at a rate of 0.1 rpm/s. Each mouse underwent three test trials. The experiment was considered complete after the mouse slipped off the path or when 5 min had passed. The motion test data is taken as the average of the three test drop times.

### 2.8 Statistical analysis

All the data were analyzed using Graph Pad Prism 7.0 (GraphPad Software) and presented as the mean ± standard error of the mean (SEM). Data sets were tested for normality of distribution with the Shapiro–Wilk test and all data exhibited normal distribution. *T*‐test was performed for comparisons between the two groups. A one‐way ANOVA test was applied for comparisons among multiple groups, followed by either Fisher's LSD or Tukey's post hoc test. If the *p* < 0.05, the difference was considered significant.

## 3 RESULTS

### 3.1 Identification of Significantly Differentially Expressed Proteins

**FIGURE 1:**
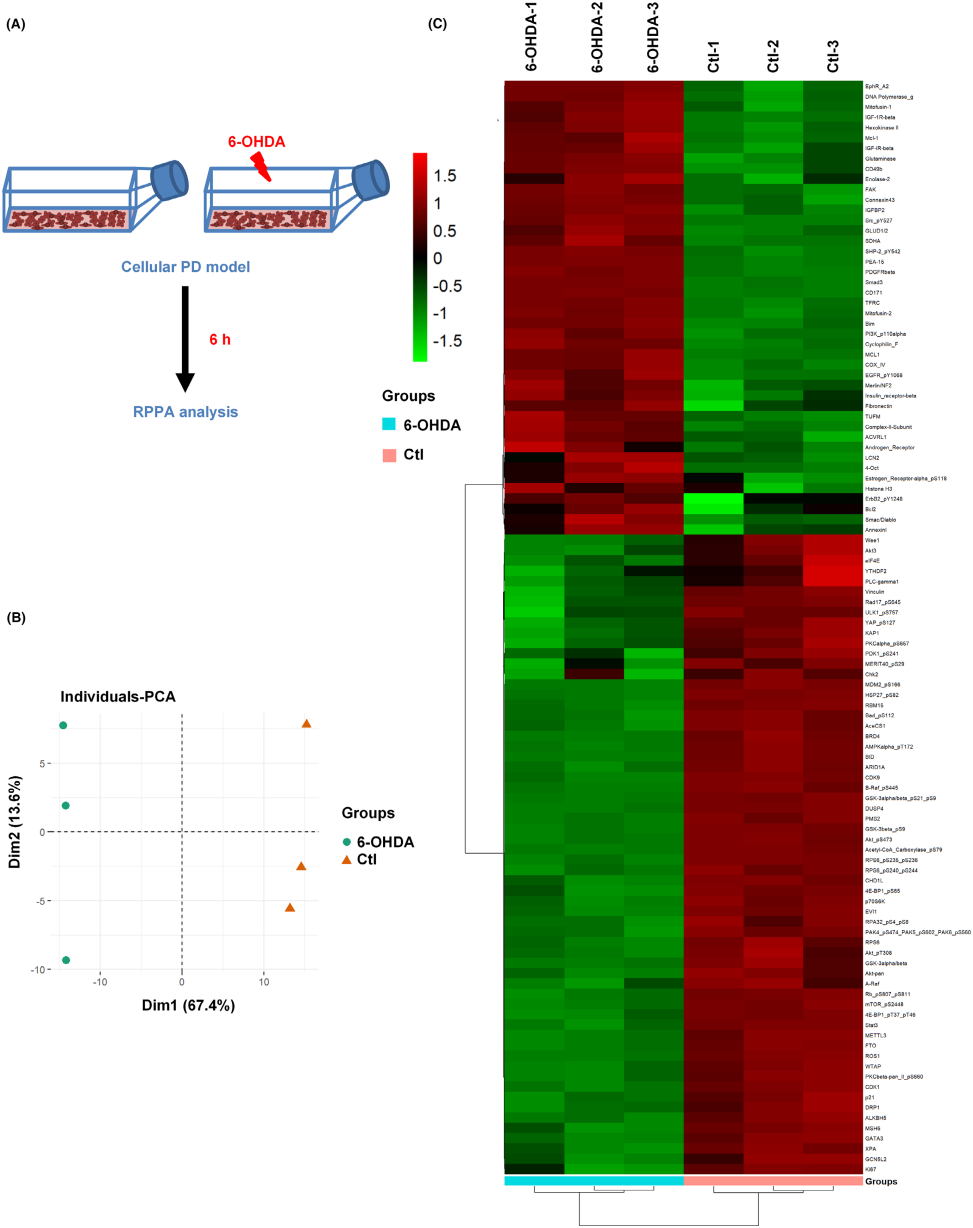
Reverse phase protein array (RPPA) analysis of 6‐OHDA induced cellular Parkinson's disease (PD) model. (A) The experiment is divided into two groups, one is the 6‐OHDA group and the other is the Ctl group. SH‐SY5Y cells were treated with 6‐OHDA for 6 h, then collected for RPPA analysis. (B) In the principal component analysis diagram, each point represents a single sample. Proteins with similar significantly differentially expressed (SDE) are distributed together, while proteins with different SDE are scattered. (C) Based on SDE proteins, clustering analysis was shown in Heatmap. Each column represents one group of samples (abscissa is the sample information) and each row represents one protein (ordinate is the significant differentially expressed protein), where red areas represent up‐regulation of protein expression, and green areas represent down‐regulation of protein expression.

6‐OHDA is a neurotoxin to dopaminergic neurons commonly used to induce experimental parkinsonism.^49^, ^50^ To explore the potential biomarkers of PD, we established a cellular PD model and collected cells for RPPA analysis (Figure 1A). The principal component analysis (PCA) plot (Figure 1B) indicated that the first principal component (67.4%) separated the Ctl and 6‐OHDA samples, revealing that the proteins in these two groups are different. Expression values of the Ctl and 6‐OHDA samples have been visualized as heatmaps (Figure 1C, *p* < 0.05). There were 305 differentially expressed proteins between the Ctl and 6‐OHDA‐treated groups, among which there were 106 significantly differentially expressed (SDE) proteins, including 44 upregulated proteins and 62 downregulated proteins (Table S1). Among them, Akt_pS473_, PDK1_ps241_, mTOR_pS2448_, and ErbB2_pY‐1248_ play important roles in the pathogenesis of PD.^51^, ^52^, ^53^, ^54^

### 3.2 GO and KEGG Analysis of SDE Proteins

**FIGURE 2:**
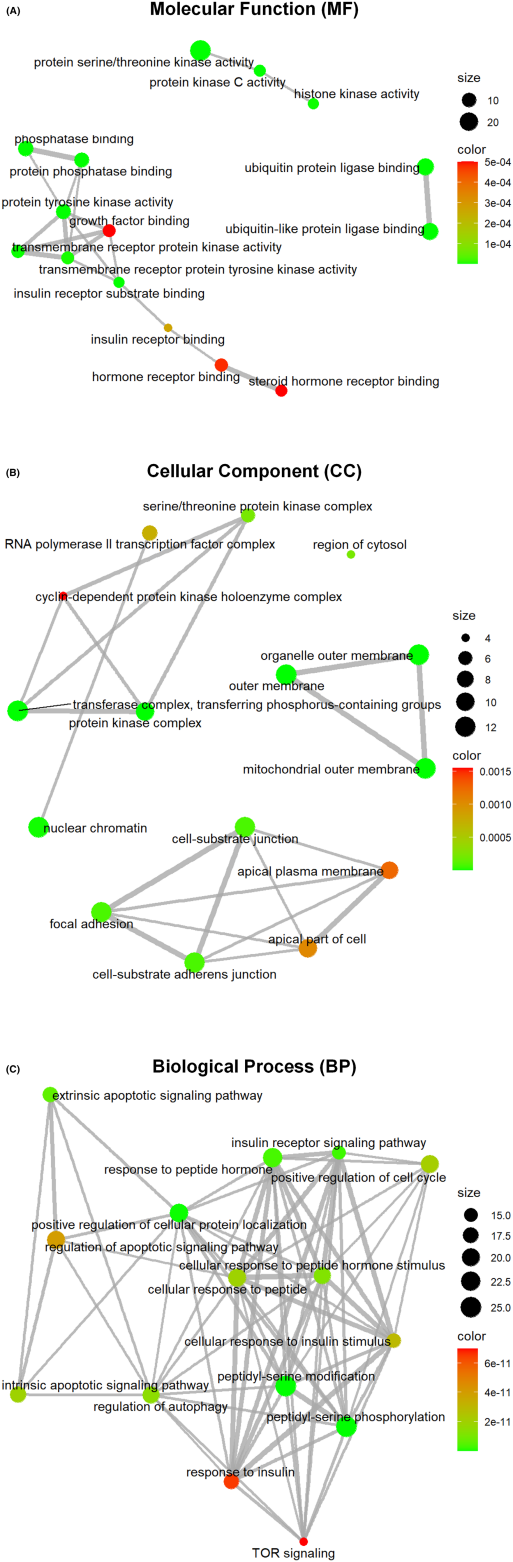
GO enrichment analysis of SDE proteins. It divided the function of SDE proteins into three parts: (A) molecular function (MF), (B) cellular component (CC), and (C) biological process (BP). The results of the GO analysis showed that the process involved 15 MF, 15 CC, and 15 BP. The size of the node represents the number of enriched SDE proteins. The *p* value is represented by a color scale, where the statistical significance increases as red turns to green.

GO analysis was performed to investigate these SDE proteins' functional properties. Among the 45 GO terms which derived the features in the optimal feature set, 15 terms turned out to describe molecular functions (MFs), 15 terms are associated with cellular components (CCs), and 15 terms are involved in biological processes (BPs) (Figure 2). In the GO analysis, SDE proteins that are categorized in the MF ontology are mainly annotated to the terms response to transmembrane receptor protein tyrosine kinase activity, growth factor binding, et al. The CC terms were apical part of the cell, cell‐substrate junction, focal adhesion, et al. The BP annotated terms primarily consisted of response to insulin, TOR signaling, peptidyl‐serine phosphorylation, et al.

**FIGURE 3:**
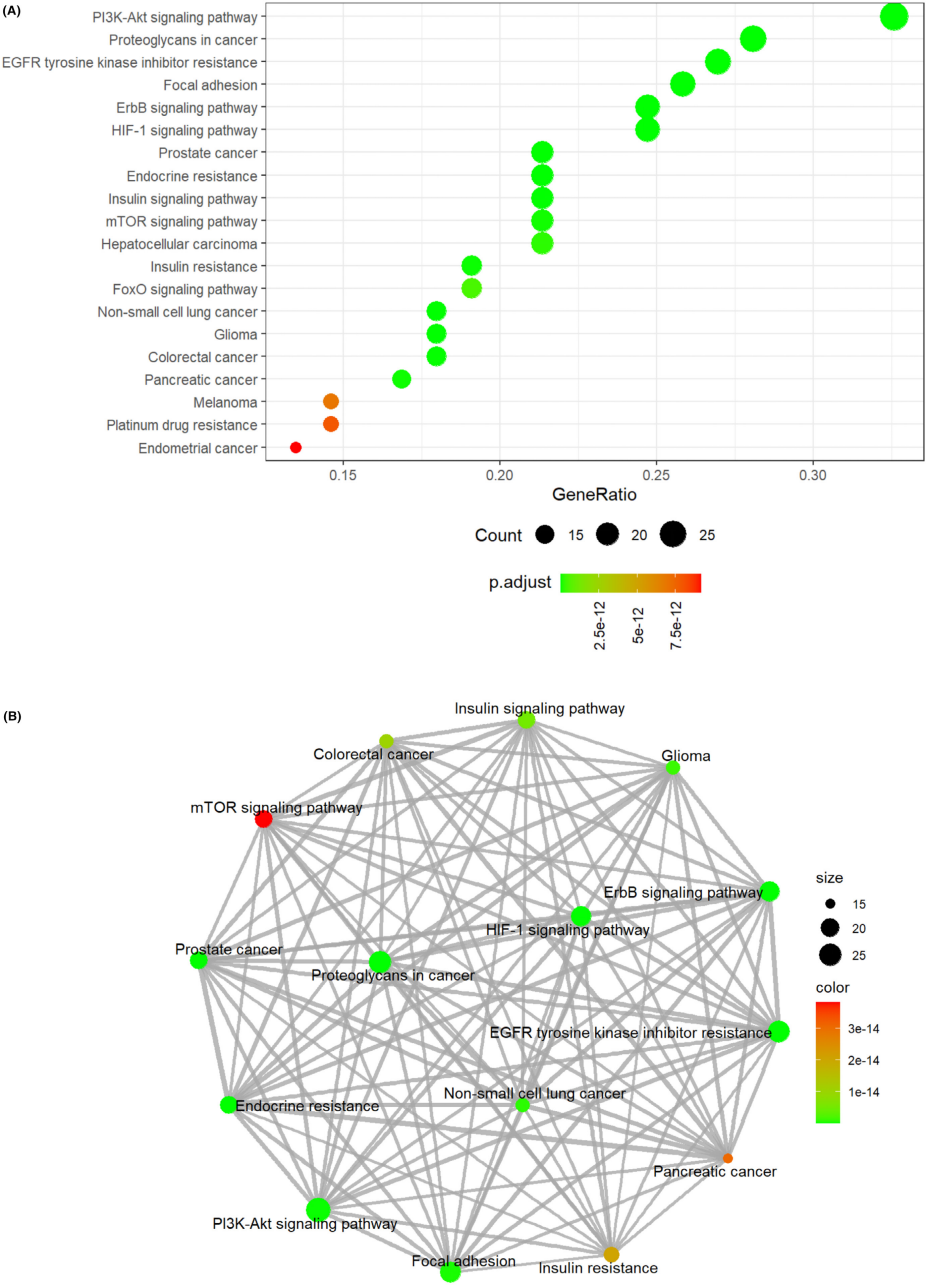
The top 20 pathways were obtained from KEGG pathway analysis. (A) The X‐axis is the ratio of the number of SDE proteins enriched in the pathway to the number of proteins annotated in the pathway. Y‐axis shows the KEGG pathway items. (B) Pathway relationship network. The nodes represent pathways enriched by SDE proteins. The size of the dots in the graph indicates the number of SDE proteins enriched in the pathway. The color indicates the significant *p* value of the pathway.

To get a more specific understanding of the relevant pathways responsible for the SDE proteins, KEGG analysis was performed. The top 20 significant enrichment pathways with the highest protein counts were presented in Figure 3, implying that these signaling pathways regulate PD. The main KEGG pathways were P13K Akt signaling pathway, EGFR tyrosine kinase inhibitor resistance, focal adhesion, HIF‐1 signaling pathway, and ErbB signaling pathway. Among them, the ErbB signaling pathway has been reported to play a pivotal role in PD. Considering findings obtained from the RPPA study, including top SDE proteins (with post‐translational modifications), GOs, and KEGG pathways, ErbB2_pY‐1248_ possibly acts as a potential biomarker of PD.

### 3.3 Validation of ErbB2_pY_ _‐1248_ as a biomarker candidate for PD

**FIGURE 4:**
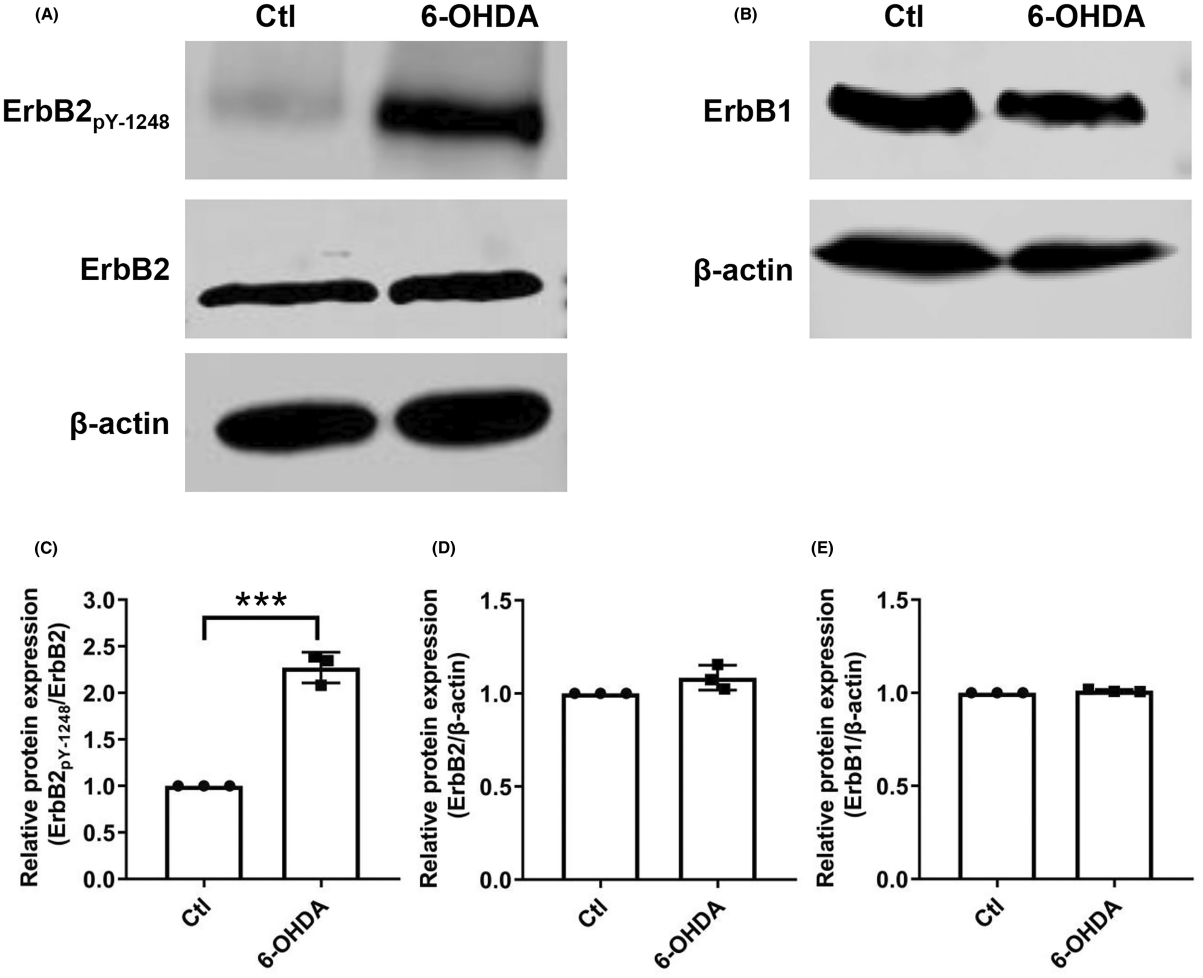
Western blot analysis of ErbB2_pY‐1248_, ErbB2, and ErbB1 in cellular PD model. (A, C) Representative images of western blot of ErbB2_pY‐1248_, ErbB2, and ErbB1 in extracted protein lysates from SH‐SY5Y cells treated with 6‐OHDA for 6 h. (B, D) Grayscale value analysis of the immunoblots. The band intensity of ErbB2_pY‐1248_ and ErbB1 were normalized to ErbB2 and β‐Actin, respectively. It is presented as mean ± SEM. ***p < 0.001 vs. Ctl.

To verify the important roles of ErbB2_pY‐1248_ in PD revealed by RPPA analysis, we carried out a western blot to test the protein expression of ErbB2_pY‐1248_, ErbB2, and ErbB1. There was a significant increase in the protein expression of ErbB2_pY‐1248_ in the 6‐OHDA group when compared to the Ctl group (Figure 4A,C). The total protein levels of ErbB2 and ErbB1 showed no apparent change in the 6‐OHDA group compared with that of the Ctl (Figure 4A,B,D,E). The original images of each cropped gel/blot are provided in Figure S1.

### 3.4 Further verification of ErbB2_pY‐1248_ by zebrafish and mouse model

**FIGURE 5:**
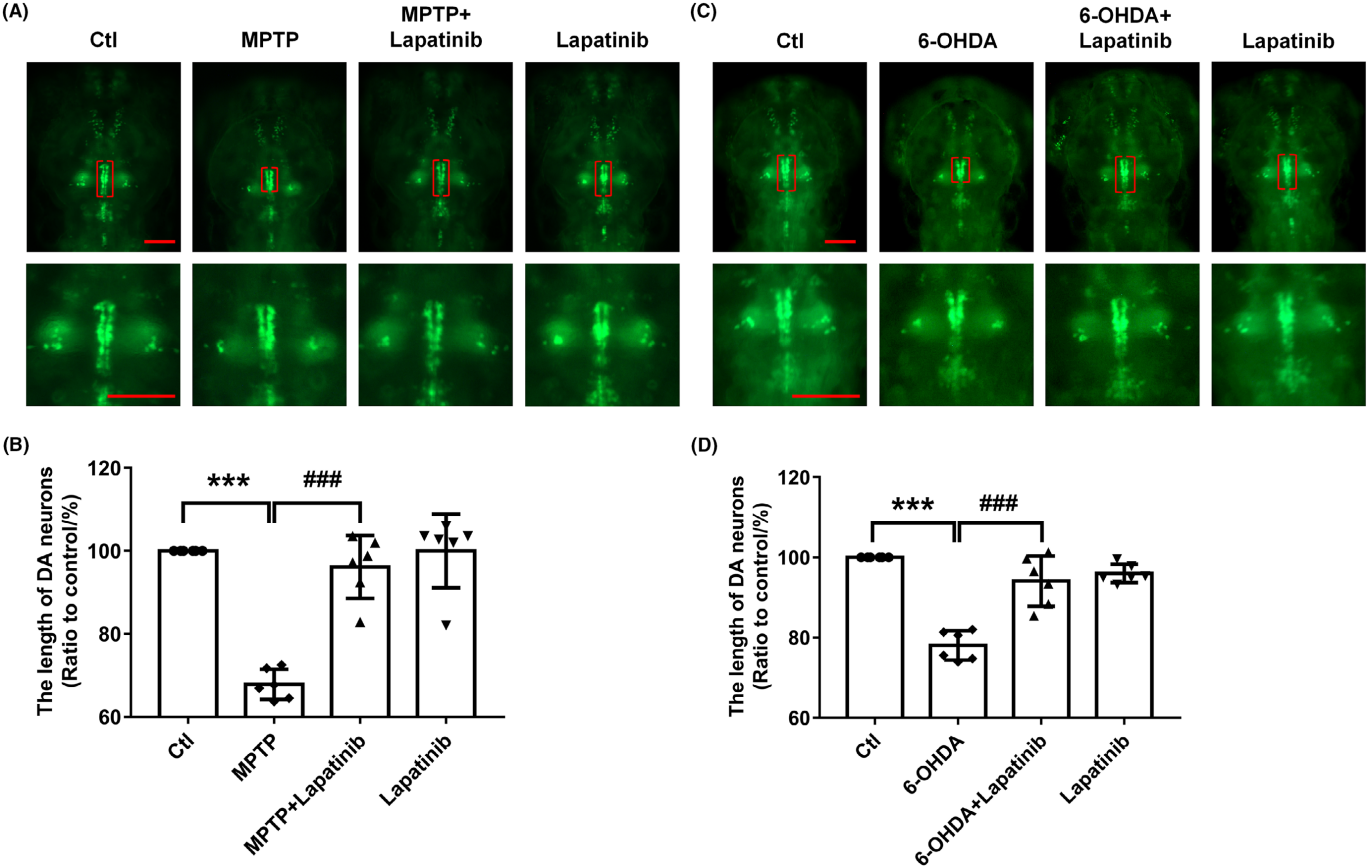
Effects of lapatinib on the length of dopaminergic neurons in zebrafish PD model. (A, C) Representative fluorescence images of *vmat2*: *GFP* zebrafish. Dopaminergic neurons are indicated in red brackets and display enlarged images to improve the visualization of dopaminergic neurons morphology. Scale bar, 100 μm. (B, D) Statistical analysis of the length of dopaminergic neurons from each experimental group (*n* = 6, ***p < 0.001 vs. Ctl; ^###^ *p* < 0.001 vs. MPTP or 6‐OHDA).

By using the zebrafish PD model, we further verify the potential of ErbB2_pY‐1248_ as a PD biomarker. Lapatinib, a tyrosine kinase inhibitor of ErbB2, was used in this study. Zebrafish dopaminergic neurons are entirely developed by 96 hpf.^55^ To investigate whether lapatinib reverses PD‐like symptoms in zebrafish, we assessed the dopaminergic neurons. Compared with the Ctl group, the dopaminergic neuron length in the MPTP‐treated group was significantly decreased, while lapatinib reversed this decrease to the normal (Figure 5A,B). Similarly, we found that 6‐OHDA exposure significantly decreased the length of dopaminergic neurons, while lapatinib and 6‐OHDA co‐treatment revealed a remarkable increase (Figure 5C,D).

**FIGURE 6:**
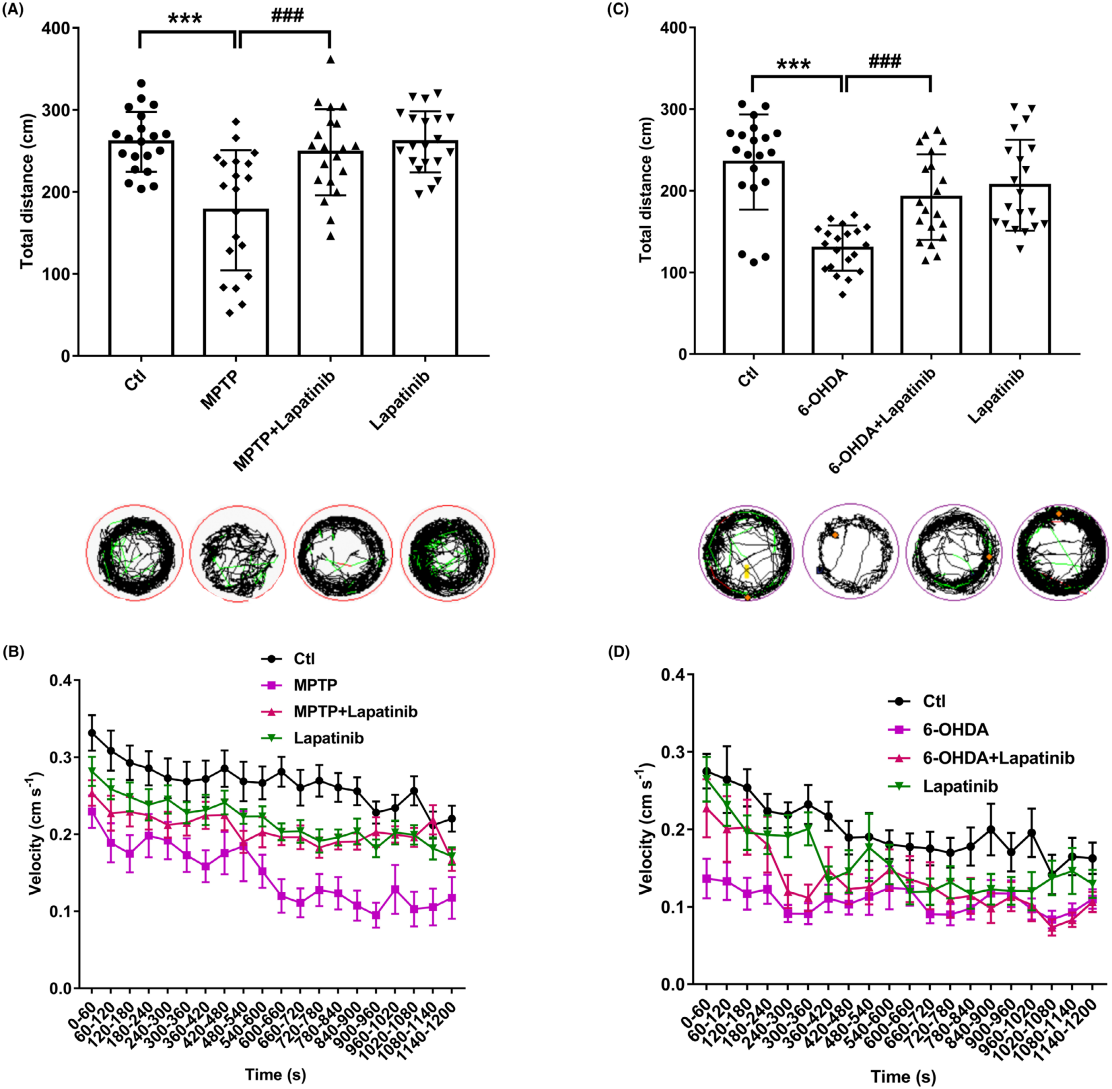
Effects of lapatinib on locomotion impairments in zebrafish PD model. (A, C) Total distance traveled by zebrafish. Red, green, and black lines depict fast, medium, and slow movement. ***p < 0.001 vs. Ctl group; ^###^ *p* < 0.001 vs. MPTP or 6‐OHDA, motion track (*n* = 20). (B, D) The average speed of zebrafish was taken every 60 s from different groups.

MPTP could induce locomotor retardation manifested in zebrafish as decreased velocity.^56^, ^57^ As expected, compared with the Ctl group, the total distance and average velocity traveled by zebrafish in the MPTP‐treated group were significantly decreased, which was consistent with the previous findings.^19^ In contrast, lapatinib and MPTP co‐treatment reversed this decrease, which suggested that lapatinib alleviated MPTP‐induced suppression of the locomotor capacity of zebrafish (Figure 6A,B). Similarly, the behavioral tests of zebrafish larvae at 5 dpf showed remarkable differences in the total distance and average velocity traveled between the 6‐OHDA and Ctl groups. Larval zebrafish exposed to 6‐OHDA exhibited an apparent decrease in locomotor activity, while lapatinib treatment significantly increased the total distance and average velocity moved (Figure 6C,D). In brief, lapatinib blocked the phosphorylation of ErbB2, reversing zebrafish PD‐like symptoms, which implied that ErbB2 phosphorylation is essential for PD development and progression.

**FIGURE 7:**
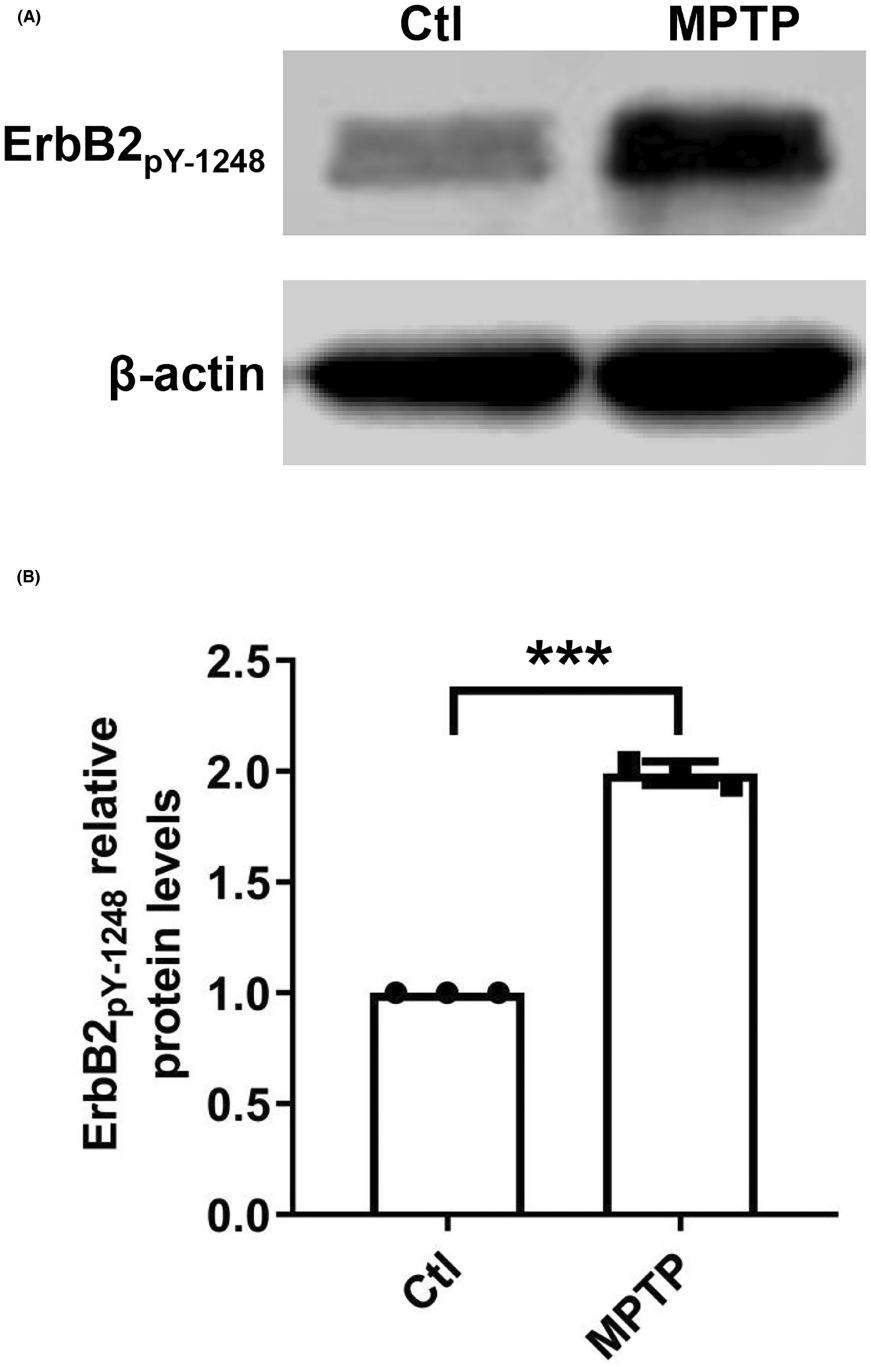
Western blot analysis of ErbB2_pY‐1248_ in mouse PD model. (A) Representative images of western blot of ErbB2_pY‐1248_ in the mouse brain tissues. (B) Grayscale value analysis of the immunoblots. The band intensity of ErbB2_pY‐1248_ was normalized to β‐Actin. ***p < 0.001 vs. Ctl.

To further confirm the ErbB2_pY‐1248_ role in PD, we investigated the protein expression of ErbB2_pY‐1248_ in the brain of the mouse PD model. MPTP‐induced PD mouse exhibited a dramatic decrease in the latency to fall in the PD group (30.06 ± 5.59) compared with the Ctl group (121.44 ± 15.29). In addition, the protein expression of ErbB2_pY‐1248_ significantly increased in the mouse PD model (Figure 7A,B). The original images of each cropped gel/blot are provided in Figure S2.

## 4 DISCUSSION

PD is the second most common neurodegenerative disease after Alzheimer's disease, affecting approximately 1% of the global population.^58^, ^59^ PD is now considered a systemic disease more than a complex motor disorder because its non‐motor symptoms usually precede clinical motor signs.^60^ There are still no effective strategies to stop the progression of this disease. Therefore, there is an urgent need to explore new effective therapeutic targets, especially in the early stages of PD. Although various studies have been conducted to discover potential PD candidate biomarkers, only a few biomarkers have been translated into clinical practice.^61^, ^62^

RPPA is an antibody‐based proteomics technique suitable for analyzing protein levels and post‐translational modifications, including phosphorylation.^63^, ^64^, ^65^ It has been widely used to discover disease biomarkers and study molecular mechanisms.^45^, ^66^, ^67^ Although RPPA is commonly used in cancer research, its advantages of detecting several hundred proteins in a cost‐effective, sensitive, and high‐throughput manner expand its usage. For example, the RPPA platform has the potential for translational applications by discovering and validating epigenetic states as therapeutic targets and biomarkers.^68^ Here, we used RPPA technology to predict PD biomarkers for the first time. Our study identified 106 SDE proteins, including ErbB2_pY‐1248_. ErbB2 signaling contributes to the pathogenesis of PD.^42^, ^69^, ^70^ It has been reported that ErbB signaling displays a neurotrophic and neuroprotective role in dopaminergic neurons in PD animal models.^71^ In addition, it has been shown that ErbB2 is a hub protein of the PD‐specific PPI network, suggesting that ErbB2 is a potential regulator of PD.^53^

The GO analysis revealed that the SDE proteins are significantly associated with transmembrane receptor protein tyrosine kinase activity, intrinsic apoptotic signaling pathway, mitochondrial outer membrane, and growth factor binding. In addition, KEGG analysis revealed that the SDE proteins were mainly enriched in the P13K Akt signaling pathway, EGFR tyrosine kinase inhibitor resistance, HIF‐1 signaling pathway, and ErbB signaling pathway. All findings from the above GO and KEGG analyses are consistent with the previous PD‐related studies,^51^, ^72^, ^73^ suggesting that the RPPA technology used here is reliable. Our study provides a promising way to explore biomarkers of neurodegenerative diseases. We found that the ErbB2 signaling is a critical pathway involved in PD, which was in line with the reported studies.^43^, ^74^, ^75^ Accordingly, western blot results showed that ErbB2_pY‐1248_ protein expression was dramatically upregulated in the cellular PD model. In contrast, there was no apparent change in the protein expression of ErbB1 and ErbB2. It has been reported that ErbB1 and ErbB2 are mainly expressed in the heart and cancerous tissues, with low expression in mouse brains.^76^ We found that ErbB2_pY‐1248_ protein expression was significantly higher in the mouse PD model compared with the Ctl group, which further confirmed that ErbB2_pY‐1248_ could be the biomarker of PD. Phosphorylation of proteins acting as a disease biomarker has been widely reported.^77^, ^78^, ^79^, ^80^, ^81^, ^82^ For example, plasma tau phosphorylated at threonine 217 (p‐tau217) and 181 (p‐tau181) is associated with Alzheimer's disease pathology.^80^ Site‐specific phosphorylation and caspase cleavage of glial fibrillary acidic protein (GFAP) are new biomarkers of Alexander's disease.^81^ It has been reported that the enhanced phosphorylation of c‐Jun in response to cisplatin treatment could be a promising biomarker for the efficacy of cisplatin in patients.^82^ All the above findings imply that the phosphorylation (at tyrosine 1248) of ErbB2 could be a biomarker for PD.

The intracellular carboxyl terminus of ErbB2 contains tyrosine residues that serve as phosphorylation sites for the kinase. ErbB2 contains five major tyrosine autophosphorylation sites. PY1248 is one of the C‐terminal sites phosphorylated before downstream signaling occurs.^83^, ^84^, ^85^ In the case of overexpression, ErbB2_pY‐1248_ is the most potent site because it is constitutively activated due to ErbB2 homodimerization.^86^ In addition, among the multiple ErbB2 tyrosine phosphorylation sites, pY1248 has been documented to be biologically meaningful and clinically significant. Cell culture data previously showed that tyrosine phosphorylation at ErbB2_pY‐1248_ plays a role in the negative regulation of ErbB2‐coupled signaling.^87^ Phosphorylation at the 1248 site prompts ErbB2 to connect with the Ras–Raf‐MAPK signaling pathway.^88^ Therefore, we predicted that ErbB2_pY‐1248_ might be a biomarker for the early diagnosis of PD.

Zebrafish are widely used in the study of PD pathogenesis because of their rapid life cycle and close genetic similarity to humans. MPTP or 6‐OHDA exposure is a well‐established animal PD model commonly used in zebrafish to induce PD.^89^, ^90^ Zebrafish exhibit injured dopaminergic neurons and altered locomotor activity when exposed to MPTP or 6‐OHDA.^91^, ^92^ In the current study, zebrafish treated with MPTP or 6‐OHDA induced an apparent decrease in the length of dopaminergic neurons and locomotor activity, which were consistent with the previous research that MPTP or 6‐OHDA could induce PD‐like behavior in a zebrafish model.^19^, ^93^ Notably, lapatinib could reverse this decrease to the normal level. These findings further demonstrate that phosphorylation of ErbB2 plays an important role in the pathogenesis of PD, thus providing support that ErbB2_pY‐1248_ is a promising biomarker of PD.

Future studies are necessary to be performed to validate the sensitivity and specificity of ErbB2_pY‐1248_ as a PD biomarker and confirm its high correlation with disease development and progression. IPSCs technology is a promising tool to support the clinical application of ErbB2_pY‐1248_, which can efficiently generate iPSCs from readily available tissues, differentiating into CNS‐specific cells.^94^, ^95^, ^96^ Long‐term survival and function of dopaminergic neurons derived from autologous human iPSCs have been reported in non‐human primate models of PD.^97^, ^98^ In the future, iPSCs might be applied to PD diagnosis by using ErbB2_pY‐1248_ as a PD biomarker.

## 5 CONCLUSION

Although PD biomarker candidates have been reported, such as α‐syn, uric acid, and glutathione, biomarker applications remain limited. In this study, we found that ErbB2_pY‐1248_ is a predictive biomarker of PD by using RPPA technology and in vivo verification. It offers a new perspective on PD diagnosing and treatment, which will be essential in identifying individuals at risk of PD. In addition, this study provides new ideas for digging into biomarkers of other neurodegenerative diseases.

## AUTHOR CONTRIBUTIONS

Meng Jin: Supervision, Conceptualization, Methodology, Formal analysis, Writing‐review & editing. Ruidie Shi: Writing, Formal analysis, Visualization. Daili Gao and Baokun Wang: Visualization and Formal analysis. Ning Li: Investigation. Xia Li: Formal analysis. Attila Sik: Formal analysis, Writing‐review & editing. Kechun Liu: Investigation, Resources. Xiujun Zhang: Supervision, Resources. All the authors read and approved the final manuscript.

## FUNDING INFORMATION

This work was supported by the Jinan Talent Project for Universities (Nos. 2021GXRC106, 2021GXRC111, and 2019GXRC044). We are also grateful for grants from the Education and Industry Integration Innovation Pilot Project of Qilu University of Technology (Shandong Academy of Sciences) (Nos. 2022PY033, 2022GH022, and 2022JBZ01‐06) and Shandong High‐end Foreign Experts Recruitment Program (Nos. WST2019006 and WST2020008).

## CONFLICT OF INTEREST STATEMENT

None.

## Supporting information


Figure S1

Figure S2

Table S1

## Data Availability

The datasets generated during and/or analyzed during the current study are available from the corresponding author upon reasonable request.
