# Supplementary figures and images for: ErbB2pY ‐1248 as a predictive biomarker for Parkinson's disease based on research with RPPA technology and in vivo verification
Source: CNS Neurosci Ther. 2023 Aug 11;30(2):e14407. doi: 10.1111/cns.14407 (PMC10848095; doi:10.1111/cns.14407)

## Full unedited blot for Figure 4

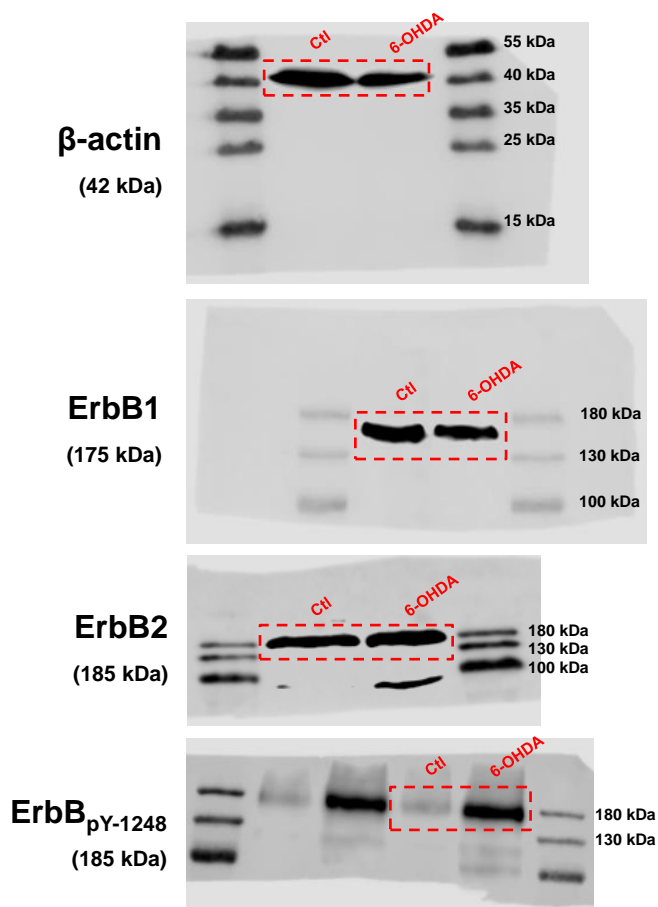

Supplement: Supplementary file 1 — Figure S1 [file CNS-30-e14407-s003.pdf]

## Full unedited blot for Figure 7

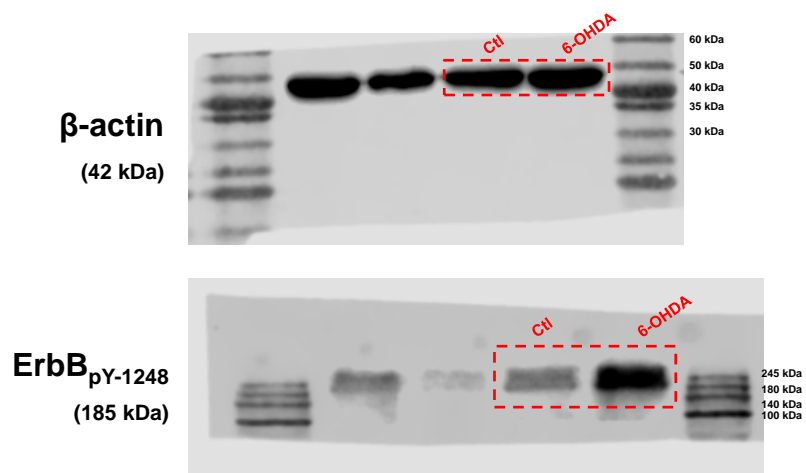

Supplement: Supplementary file 2 — Figure S2 [file CNS-30-e14407-s002.pdf]
